# Comparison of Clinical, Epidemiological, Haematological, and Biochemical Characteristics in Serologically Confirmed and Suspected Cases of Tularemia

**DOI:** 10.3390/tropicalmed10100289

**Published:** 2025-10-10

**Authors:** Nurten Nur Aydın, Murat Aydın, Ömer Karaşahin

**Affiliations:** Department of Infectious Diseases and Clinical Microbiology, Erzurum Regional Training and Research Hospital, Erzurum 25080, Turkey; murat.aydin5@saglik.gov.tr (M.A.); omer.karasahin@saglik.gov.tr (Ö.K.)

**Keywords:** tularemia, zoonotic infections, lymphadenopathy, retrospective study, MAT, Turkey

## Abstract

Background: Tularemia is a zoonotic infection caused by *Francisella tularensis*, transmitted to humans through direct contact with infected animals, arthropod bites, or by ingesting contaminated water. It commonly presents with fever, lymphadenopathy, and oropharyngeal symptoms. In Turkey, where waterborne outbreaks are frequent, tularemia remains a significant public health concern. This study aimed to compare the clinical, epidemiological, and laboratory characteristics of patients diagnosed with tularemia and those with similar clinical features but seronegative results, with the goal of identifying parameters that may assist in differential diagnosis. Methods: This retrospective study included adults (≥18 years) who presented to the Infectious Diseases Outpatient Clinic between 2016 and 2024 with suspected tularemia and were tested using a microagglutination test (MAT). Patients with a positive MAT (≥1:160) or a fourfold titre increase were classified as tularemia cases, while seronegative patients were defined as tularemia-like cases. Demographic data, clinical symptoms, epidemiological risk factors, and laboratory findings were compared between the two groups. Results: A total of 105 patients were included, 54 (51.4%) of whom were diagnosed with tularemia. The duration from symptom onset to healthcare presentation was significantly longer in tularemia cases (20.3 ± 5.7 vs. 15.7 ± 6.2 days; *p* < 0.001). Sore throat (66.7% vs. 43.1%; *p* = 0.026) and tonsillitis/pharyngitis (55.6% vs. 21.6%; *p* = 0.001) were significantly more prevalent in the tularemia group. Epidemiological risk factors, including rural residence (92.6%), animal husbandry (74.1%), agricultural activity (72.2%), and contact with lake or stream water, were significantly more prevalent among tularemia cases (all *p* < 0.001). Alanine aminotransaminase (*p* = 0.019) and C-reactive protein levels (*p* = 0.027) were significantly lower in the tularemia group. Conclusions: Tularemia cases are associated with particular epidemiological risk factors and oropharyngeal symptoms. A thorough epidemiological evaluation is crucial for diagnosis, and enhancing awareness among healthcare providers and the public may facilitate earlier recognition and management.

## 1. Introduction

Tularemia is a highly infectious zoonotic disease caused by the Gram-negative, facultative intracellular coccobacillus, *Francisella tularensis* [1]. This bacterium, for which wild rabbits, rodents, and various arthropods count as its natural reservoirs, can be transmitted to humans through direct contact with infected animals, the consumption of contaminated water and food, inhalation, or vectors [1,2]. The clinical presentations may manifest in six distinct forms, namely oropharyngeal, glandular, ulceroglandular, oculoglandular, pneumonic, and typhoidal symptoms, depending on the mode of transmission and the host’s immune status [3]. The high incidence of oropharyngeal cases in residents of Turkey indicates that contaminated water sources are a significant route of transmission, particularly in outbreak settings [4,5]. These clinical and epidemiological characteristics, including waterborne outbreaks, are notably specific to the Turkish context and may differ from patterns observed in other endemic regions.

Cases of tularemia typically arise in small outbreaks or clusters and may be asymptomatic or exhibit nonspecific symptoms, which can result in delays in diagnosis [6,7]. While full recovery is achievable with early diagnosis and suitable antibiotic treatment, delays in diagnosis can lead to treatment failure and the development of complications. Thus, a more thorough understanding of the epidemiological characteristics, risk factors, and clinical–laboratory parameters of tularemia is crucial for both individual patient management and public health. Tularemia, particularly in its oropharyngeal form, is characterised by nonspecific symptoms such as sore throat, tonsillitis, and cervical lymphadenopathy [6]. Consequently, it can be easily confused with other infectious diseases, including acute tonsillopharyngitis, acute lymphadenitis, and mononucleosis. This situation can lead to delayed diagnosis, misdiagnosis, and unnecessary antibiotic use, particularly in endemic areas. There are very few studies in the literature that comparatively evaluate the epidemiological and laboratory characteristics of cases with clinical findings similar to tularemia that have been excluded by serological tests.

The aim of this study is to compare the clinical, epidemiological, and laboratory characteristics of patients diagnosed with tularemia and those with similar clinical presentations but seronegative findings in order to identify critical parameters that may contribute to differential diagnosis.

## 2. Materials and Methods

### 2.1. Study Design

This study was conducted by retrospectively reviewing the medical records of patients who visited the Infectious Diseases Outpatient Clinic at the Erzurum Regional Training and Research Hospital from January 2016 to June 2024 and whose samples were sent for a microagglutination test (MAT) with a preliminary diagnosis of tularemia. Adult patients aged 18 years and older were included in the study. The diagnosis of tularemia was based on consistent clinical findings and sociodemographic characteristics, along with a positive serum MAT (≥1/160) or a fourfold increase in MAT titre from a second serum sample taken at least two weeks later. Patients were regarded as suspected tularemia cases if they exhibited flu-like symptoms such as fever, fatigue, and myalgia, along with sore throat, tonsillitis, and/or pharyngitis, as well as cervical lymphadenopathy. Further features that heightened suspicion included residing in rural areas and a lack of clinical improvement following antibiotics known to be ineffective against tularemia, such as beta-lactams, macrolides, or trimethoprim–sulfamethoxazole, or worsening symptoms despite these treatments. These criteria reflect the typical clinical profile of oropharyngeal tularemia in Turkey, where this form is predominant, and were therefore used as the inclusion criteria for MAT testing and case selection. Patients who met these criteria were classified as ‘tularemia cases’, while those who did not meet these criteria were classified as ‘tularemia-like cases’.

### 2.2. Study Group

Tularemia cases with regional lymphadenopathy and no primary ulcer were classified as the glandular form; patients with fever and/or a sore throat were classified under the oropharyngeal form if they exhibited tonsillitis, pharyngitis, mouth ulcers, and cervical LAP. Demographic characteristics (age, gender), clinical findings (symptoms and physical examination findings), time of presentation (time elapsed from symptom onset to outpatient presentation), epidemiological risk factors (livestock farming, rural residence, agricultural activity, drinking water source, vector contact, etc.), and laboratory parameters (complete blood count, liver enzymes, acute phase reactants, etc.) were compared.

Before starting the study, approval was obtained from our hospital’s local ethics committee (decision number: BAEK 2024/07-131) on 10 July 2024.

### 2.3. Statistical Analysis

Data analysis was performed using the IBM SPSS 23.0 statistical package programme. Descriptive statistical methods were employed to analyse the data; categorical variables are presented as percentages and counts, while continuous variables are reported as mean ± standard deviation. Student’s *t*-test was used to compare continuous variables that displayed a normal distribution, whereas the Mann–Whitney U test was utilised for data that did not follow a normal distribution. Categorical variables were analysed using the chi-squared test. A *p*-value of <0.05 was considered statistically significant.

## 3. Results

Data on 105 patients were available and included in the study. Among these, 54 (51.4%) were diagnosed with tularemia, while 51 (48.6%) had tularemia-like cases. When demographic characteristics were examined, the male-to-female ratio was higher in tularemia cases (63.0% vs. 47.1%), although no statistically significant difference was found (*p* = 0.149). The average age was comparable in both groups (33.8 ± 14.9 in tularemia cases and 32.8 ± 10.3 in the other group; *p* = 0.753). In tularemia cases, the interval between the onset of symptoms and referral to a healthcare facility was significantly longer (20.3 ± 5.7 days vs. 15.7 ± 6.2 days; *p* < 0.001).

The oropharyngeal form was identified in 59.3% of cases (*n* = 32), while the glandular form was noted in 40.7% (*n* = 22). Among the clinical symptoms, a sore throat was significantly more prevalent in cases of tularemia (66.7% vs. 43.1%; *p* = 0.026). Tonsillitis/pharyngitis symptoms were significantly more prevalent in the tularemia group (55.6% vs. 21.6%; *p* = 0.001). In our cohort, lymphadenopathy was mainly cervical, consistent with the oropharyngeal form. No patients were found to have axillary or inguinal lymphadenopathy. No statistically significant differences were found between the groups regarding other symptoms and physical examination findings (Table 1).

When evaluating epidemiological characteristics, tularemia cases were significantly associated with livestock farming (74.1% vs. 25.5%; *p* < 0.001), living in rural areas (92.6% vs. 43.1%; *p* < 0.001), and having a history of farming (72.2% vs. 29.4%; *p* < 0.001) compared to tularemia-like cases. Furthermore, a comparable disease history in the neighbourhood or village (*p* = 0.001), exposure to lake or stream water (*p* < 0.001), the presence of rodents near the home (*p* < 0.001), and direct contact with rodents (*p* = 0.013) were significantly more prevalent among patients diagnosed with tularemia. No statistically significant differences were observed between the groups concerning other epidemiological characteristics (Table 2).

When the laboratory findings were examined, alanine aminotransaminase levels were significantly lower in tularemia cases compared to tularemia-like cases (23.4 ± 14.9 vs. 27.2 ± 22.0; *p* = 0.019). C-reactive protein levels were lower in the tularemia group (19.3 ± 18.2 vs. 24.1 ± 28.1; *p* = 0.027). No significant differences were observed between the groups regarding other haematological and biochemical parameters (Table 3).

**Figure 1:**
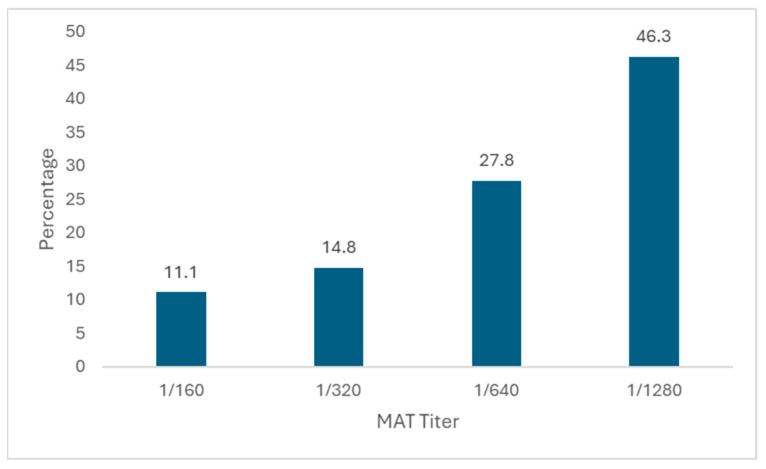
Distribution of MAT titres among patients diagnosed with tularemia.

When the distribution of MAT titres was examined in cases diagnosed with tularemia, 6 patients (11.1%) had a titre of 1/160, 8 patients (14.8%) had a titre of 1/320, 15 patients (27.8%) had a titre of 1/640, and 25 patients (46.3%) had a titre of 1/1280 (Figure 1).

## 4. Discussion

In this study, tularemia cases diagnosed based on serological tests were compared with seronegative cases that presented a clinical picture similar to tularemia. Some epidemiological and clinical differences were observed between the two groups. In Turkey, tularemia cases were more likely to have risk factors such as residing in rural areas, engaging in agriculture and animal husbandry, having contact with rodents, and being exposed to lake and river water. Symptoms such as a sore throat, tonsillitis, and pharyngitis were more prevalent in this group, and the duration between symptom onset and hospital admission was significantly longer compared to seronegative cases.

Compared to seronegative cases, the longer duration between the onset of symptoms and referral in tularemia cases, the presence of nonspecific symptoms, and the insufficient consideration of tularemia in differential diagnoses suggest delays in diagnosis. Similarly, the literature indicates that tularemia is often diagnosed late, potentially increasing the risk of complications [6,8]. In a study by Binay et al. [8], it was emphasised that diagnosis is often delayed in cases where clinical suspicion is low; consequently, beta-lactam antibiotics, in particular, are used repeatedly and unnecessarily, and surgical intervention may be required due to lymph node suppuration in advanced stages. It was also noted that this situation might create an additional burden on both patients and the health system, and it was suggested that training could be beneficial in increasing awareness among physicians and the public.

In our study, the finding that tonsillitis and pharyngitis, along with a sore throat, were more frequently observed in tularemia cases compared to seronegative patients suggests that tularemia should be considered in the differential diagnosis of patients presenting with such symptoms, particularly in endemic regions such as Turkey, where the oropharyngeal form is common. The inclusion criteria for suspected tularemia in this study were based on a combination of clinical findings that are particularly relevant in Turkey. Patients usually presented with flu-like symptoms, a sore throat, and tonsillitis or pharyngitis accompanied by cervical lymphadenopathy. The lack of response to beta-lactams, macrolides, or trimethoprim–sulfamethoxazole or the worsening of symptoms despite such treatments further reinforced clinical suspicion. Although these signs may overlap with other upper respiratory tract infections, in endemic areas such as Turkey, they strongly suggest tularemia—especially when combined with epidemiological risk factors like rural living, agriculture, animal husbandry, and contact with untreated water sources. In Turkey, this form is the most prevalent clinical manifestation of tularemia, especially in rural areas where exposure to contaminated water sources is widespread. However, in other tularemia-endemic countries, the main clinical form may differ depending on local transmission routes and epidemiological factors. It typically presents with symptoms resembling upper respiratory tract infections, including a sore throat, cervical lymphadenopathy, tonsillitis, and pharyngitis [9,10]. In our study, 67% of patients presented with a sore throat, and 56% exhibited symptoms of tonsillitis or pharyngitis. In Turkey, the oropharyngeal form is the most common clinical presentation, underscoring this clinical pattern. The literature also reinforces this fact. However, tonsillopharyngeal symptoms may complicate recognising the disease, potentially leading to delays in diagnosis [6,8,11].

Epidemiological data obtained from our study in Turkey indicate that tularemia cases frequently occur in individuals residing in rural areas, who are engaged in agriculture and animal husbandry, and who have a history of contact with rodents or potentially contaminated natural water sources, such as lake and stream water. These findings reveal the relationship between natural reservoirs and transmission routes of tularemia, emphasising the importance of rigorous epidemiological questioning in the diagnostic process within endemic areas. Similarly, rural living, contact with animals, and exposure to contaminated water are highlighted as significant risk factors for tularemia in the literature [12,13,14].

Our findings show that several epidemiological factors were significantly more common among tularemia patients compared with non-tularemia cases. Specifically, living in rural areas, engaging in agriculture and animal husbandry, contact with rodents, and exposure to contaminated natural water sources were strongly linked to tularemia. Among these, rural residence seems to be the most fundamental factor, as it involves a higher likelihood of engaging in agricultural activities, livestock breeding, and dependence on natural water sources. These factors are also interconnected, as previous studies have shown, with rural populations more likely to participate in farming and to be exposed to untreated water or rodent reservoirs [9,15,16]. In the Turkish context, where oropharyngeal tularemia is prevalent, contact with contaminated natural waters and rodents should be considered especially significant as primary factors in waterborne transmission [10,14]. These findings emphasise the importance of thorough epidemiological history-taking in endemic areas to differentiate tularemia from other conditions with similar clinical features.

In a comparison of the laboratory parameters of patients diagnosed with tularemia and seronegative patients exhibiting similar clinical symptoms, alanine aminotransferase levels were found to be significantly lower in the tularemia group. This may be attributed to the fact that tularemia might demonstrate a localised course of infection that does not significantly elevate liver enzymes. Although alanine aminotransferase levels were statistically lower in tularemia patients compared with non-tularemia cases, the mean values in both groups (23.4 U/L vs. 27.2 U/L) remained within the normal reference range. Hence, this difference is unlikely to be clinically significant and should be regarded as a minor variation rather than a diagnostic marker. Similarly, C-reactive protein levels were found to be significantly lower in the tularemia group. These findings suggest that the systemic inflammatory response may be more limited in certain cases of tularemia, and they also reveal that inflammation markers are not diagnostic. There was no statistically significant difference between the groups regarding other parameters such as white blood cells, aspartate aminotransferase, platelet count, albumin, and sodium levels. Similarly, it has been reported in the literature that laboratory findings in cases of tularemia are often nonspecific, with mild-to-moderate increases in inflammatory markers such as CRP and sedimentation rate being observable [17,18]. Therefore, although laboratory tests may assist in diagnosing patients with suspected tularemia, they are not conclusive on their own and should be evaluated alongside epidemiological history and clinical findings.

This study has several limitations. The retrospective design may have led to some clinical or epidemiological data being incompletely recorded. Furthermore, the final clinical diagnoses of seronegative cases presenting with tularemia-like symptoms were not analysed. This increases the heterogeneity of the compared groups and limits the interpretation of the findings. The interpretation of MAT results has certain limitations. False negative results may occur when patients present during the early stage of illness before a detectable antibody response develops. In our cohort, follow-up MAT testing was conducted in a subset of initially seronegative patients who did not show clinical improvement, and these repeat tests also remained negative. Furthermore, some MAT-negative patients improved with antibiotics that are not effective against tularemia or with only supportive care, indicating possible alternative diagnoses. Conversely, although false positive MAT results are theoretically possible in endemic regions due to the persistence of antibodies, the likelihood of this was low in our study, as MAT was only performed on symptomatic patients with compatible clinical presentations. Nevertheless, a reliance on serology means that both false negative and false positive results remain possible, which is an important limitation of our study. The MAT is widely regarded as the standard serological test for tularemia, with reported sensitivity and specificity generally ranging from 90% to 100% in various studies [19,20]. However, cross-reactivity with other Gram-negative bacteria, such as Brucella spp., has been identified, and false-negative results may occur in the early phase of the disease. These limitations emphasise that the distinction between tularemia and tularemia-like cases, although based on MAT results in our study, should be interpreted with caution. Furthermore, this study is single-centred and comprises data from only one geographical region, which restricts the generalisability of the results.

## 5. Conclusions

In conclusion, this study demonstrated that tularemia cases were more frequently linked to specific epidemiological risk factors—such as residing in rural areas, involvement in animal husbandry, contact with rodents, and exposure to contaminated water—as well as clinical manifestations of oropharyngeal involvement, including a sore throat and tonsillitis/pharyngitis. Additionally, the longer interval between symptom onset and hospital admission in this group suggests potential delays in diagnosis due to the nonspecific nature of early symptoms. These findings emphasise the importance of conducting thorough epidemiological assessments and considering tularemia in the differential diagnosis of patients presenting with oropharyngeal symptoms in endemic regions such as Turkey. Raising awareness among healthcare professionals and the public is essential to facilitate the timely recognition and diagnosis of the disease.

## Figures and Tables

**Table 1:** A comparison of clinical and demographic features in tularemia and tularemia-like cases.

	Tularemia-like Cases *n* = 51 *n* (%)	Tularemia Cases *n* = 54 *n* (%)	*p*-Value
Sex			0.149
Female	27 (52.9%)	20 (37.0%)	
Male	24 (47.1%)	34 (63.0%)	
Mean age (years) ± standard deviation	32.8 ± 10.3	33.8 ± 14.9	0.753
Time from symptom onset to outpatient admission (days)	15.7 ± 6.2	20.3 ± 5.7	<0.001
Symptoms			
Sore throat	22 (43.1%)	36 (66.7%)	0.026
Oral ulcer	3 (5.9%)	5 (9.3%)	0.717
Fatigue	27 (52.9%)	36 (66.7%)	0.217
Fever	15 (29.4%)	22 (40.7%)	0.312
Myalgia/arthralgia	16 (31.4%)	16 (29.6%)	1.000
Loss of appetite	21 (41.2%)	26 (48.1%)	0.602
Nausea/vomiting	5 (9.8%)	6 (11.1%)	1.000
Abdominal pain	6 (11.8%)	4 (7.4%)	0.519
Diarrhoea	0	1 (1.9%)	1.000
Lymph node enlargement or pain	37 (72.5%)	45 (83.3%)	0.272
Eye redness/swelling	3 (5.9%)	5 (9.3%)	0.717
Skin ulcer or lesion	4 (7.8%)	1 (1.9%)	0.197
Skin erythema or rash	6 (11.8%)	4 (7.4%)	0.519
Findings			
Fever	11 (21.6%)	20 (37.0%)	0.128
Tonsillitis/pharyngitis	11 (21.6%)	30 (55.6%)	0.001
Oral mucosal lesion	2 (3.9%)	5 (9.3%)	0.438
Lymphadenopathy	40 (78.4%)	49 (90.7%)	0.138

**Table 2:** A comparison of epidemiological features between tularemia and tularemia-like cases.

Epidemiological Feature	Tularemia-like Cases *n* = 51 *n* (%)	Tularemia Cases *n* = 54 *n* (%)	*p*-Value
History of animal husbandry	13 (25.5%)	40 (74.1%)	<0.001
Living in a rural area	22 (43.1%)	50 (92.6%)	<0.001
History of agricultural activity	15 (29.4%)	39 (72.2%)	<0.001
Similar illness in household members	2 (3.9%)	11 (20.4%)	0.024
Similar illness in the neighbourhood or village	6 (11.8%)	23 (42.6%)	0.001
Use of well water	0 (0.0%)	5 (9.3%)	0.077
Use of spring water	1 (2.0%)	3 (5.6%)	0.655
Use of public fountain water in village/neighbourhood	5 (9.8%)	5 (9.3%)	0.807
Use of municipal tap water	45 (88.2%)	41 (75.9%)	0.166
Contact with lake or stream water	0 (0.0%)	17 (31.5%)	<0.001
Unprotected contact with wild game animals	0 (0.0%)	4 (7.4%)	0.118
Presence of rodents around the home	0 (0.0%)	14 (25.9%)	<0.001
Direct contact with rodents	0 (0.0%)	7 (13.0%)	0.013
History of tick exposure	0 (0.0%)	1 (1.9%)	1.000
Outdoor activities (picnicking, hunting, sports, etc.)	17 (33.3%)	25 (46.3%)	0.248

**Table 3:** A comparison of laboratory parameters between tularemia and tularemia-like cases.

Laboratory Parameter	Tularemia-like Cases (*n* = 51) Mean ± SD	Tularemia Cases (*n* = 54) Mean ± SD	*p*-Value	Reference Range
White blood cell count (/µL)	8911 ± 3027	9505 ± 3281	0.075	4000–11,590
Neutrophil count (/µL)	5472 ± 2396	5919 ± 2626	0.977	2100–8890
Haemoglobin (g/L)	14.3 ± 1.7	14.2 ± 1.7	0.394	12.2–15.9
Platelet count (/µL)	312,314 ± 91,739	326,426 ± 95,024	0.111	152,000–383,000
Albumin (g/L)	42.5 ± 3.5	42.1 ± 3.9	0.111	32–48
Alanine aminotransferase (U/L)	27.2 ± 22.0	23.4 ± 14.9	0.019	7–40
Aspartate aminotransferase (U/L)	21.9 ± 13.7	22.4 ± 11.1	0.074	0–34
Sodium (mEq/L)	139.7 ± 2.1	139.4 ±2.9	0.520	136–145
Total bilirubin (mg/dL)	0.6 ± 0.4	0.6 ± 0.4	0.865	0.2–1.1
Erythrocyte sedimentation rate (mm/h)	26.7 ± 14.7	28.7 ± 15.1	0.054	2–20
C-reactive protein (mg/L)	24.1 ± 28.1	19.3 ± 18.2	0.027	0–5

## Data Availability

The datasets used and/or analyzed during the current study are available from the corresponding author upon reasonable request.
